# Chiropractic and Spinal Manipulation Therapy on Twitter: Case Study Examining the Presence of Critiques and Debates

**DOI:** 10.2196/publichealth.5739

**Published:** 2016-09-16

**Authors:** Alessandro R Marcon, Philip Klostermann, Timothy Caulfield

**Affiliations:** ^1^ Health Law Institute Department of Law University of Alberta Edmonton, AB Canada; ^2^ The School of Computer Science Carleton University Ottawa, ON Canada

**Keywords:** spinal manipulation, manipulation therapy, chiropractic, alternative medicine, Twitter, social media, infodemiology

## Abstract

**Background:**

Spinal manipulation therapy (SMT) is a popular though controversial practice. The debates surrounding efficacy and risk of SMT are only partially evident in popular discourse.

**Objective:**

This study aims to investigate the presence of critiques and debates surrounding efficacy and risk of SMT on the social media platform Twitter. The study examines whether there is presence of debate and whether critical information is being widely disseminated.

**Methods:**

An initial corpus of 31,339 tweets was compiled through Twitter’s Search Application Programming Interface using the query terms “chiropractic,” “chiropractor,” and “spinal manipulation therapy.” Tweets were collected for the month of December 2015. Post removal of tweets made by bots and spam, the corpus totaled 20,695 tweets, of which a sample (n=1267) was analyzed for skeptical or critical tweets. Additional criteria were also assessed.

**Results:**

There were 34 tweets explicitly containing skepticism or critique of SMT, representing 2.68% of the sample (n=1267). As such, there is a presence of 2.68% of tweets in the total corpus, 95% CI 0-6.58% displaying explicitly skeptical or critical perspectives of SMT. In addition, there are numerous tweets highlighting the health benefits of SMT for health issues such as attention deficit hyperactivity disorder (ADHD), immune system, and blood pressure that receive scant critical attention. The presence of tweets in the corpus highlighting the risks of “stroke” and “vertebral artery dissection” is also minute (0.1%).

**Conclusions:**

In the abundance of tweets substantiating and promoting chiropractic and SMT as sound health practices and valuable business endeavors, the debates surrounding the efficacy and risks of SMT on Twitter are almost completely absent. Although there are some critical voices of SMT proving to be influential, issues persist regarding how widely this information is being disseminated.

## Introduction

Despite its popularity, spinal manipulation therapy (SMT) remains a controversial practice in some circles [1,2]. While there are studies that suggest it is effective for some conditions—such as lower back pain [3-5]—other studies have questioned its clinical value or have found it to be no more effective than other approaches [6-11]. The issue of risk has also stirred debates. While some believe the concerns are overstated [12-15], other commentators point to possible serious health risks associated with treatment [16-23]. Complications are also evident in terms of establishing and evaluating the safety culture of SMT [24]. Further adding to the debate is the fact that many chiropractic clinics make claims about the health benefits of SMT for ailments for which there is little to no evidence such as for the treatment of attention deficit hyperactivity disorder (ADHD), asthma, and the boosting of the immune system [25-28]. Although data are uncertain on exactly how many people are treated for the mentioned conditions, these kinds of marketing claims are common and have created policy challenges throughout the world [26,28-30].

In this study, we explore how SMT and related controversies are addressed on social media. A growing body of literature has considered the impact and growing significance of social media, such as Twitter, as a source of health information for the general public [31-34]. Exactly how health information gets disseminated and how people are affected by that information, however, remains complex even though there seems to be little doubt that social media play an increasingly significant role [35,36]. On one hand, it has been noted that social media can function as a democratic, information-disseminating tool which increases the exposure to new information and diverse perspectives [37]. On the other hand, some recent studies have demonstrated how online social networks can come to be structured on the basis of social homophily [38], whereby individuals increasingly and primarily interact with others similar to themselves [39-41]. As a result, studies have shown that using social media can limit the diversity of one’s news [42,43] as well as create political polarization over contentious issues, especially on Twitter [44-46].

Although studies show that social media can expose individuals to novel information and diverse opinions [40,43,47], it also has the potential to create “filter bubbles” or “echo chambers,” structured with ever-increasing personalization algorithms, in which one’s views and perspectives are more often reinforced than called into question [48-50]. Because having limited access to varying perspectives can play a role in augmenting validation and confirming potential bias despite the presence of contradictory information, [51-53] questions are raised as to how positions on a health topic such as SMT are formed, held, reinforced, and contested. Understanding how chiropractic and SMT is portrayed on Twitter, therefore, will provide insight into both the salience of the efficacy or risk debate, the public understanding and awareness of the associated controversies, and the manner in which health information is disseminated.

## Methods

In order to capture public discussions about SMT (eg, “spinal manipulation therapy” is a technical term unlikely to be used by the public on Twitter, particularly given the 140 character limit), we used broad search criteria including the terms “chiropractic,” “chiropractor,” and “spinal manipulation” on Twitter’s Search API. As written on Twitter’s frequently asked questions, API stands for “Application Programming Interface” and “is a defined way for a program to accomplish a task, usually by retrieving or modifying data” [54]. In practical terms, Twitter provides the programming code structuring their media, which allows users to design and build software that interacts with Twitter and its data. Our team designed a program that interacts with Twitter’s API search engine allowing us to input search terms, then collect and store all tweets in which those terms appeared. Although very large datasets can be built through Twitter’s API, it is not possible to obtain every tweet matching the search criteria. Instead, tweets deemed most “relevant” are provided by Twitter.

An initial search revealed over 3,000,000 tweets, so we selected the most recent full month: December 2015, and created 3 corpora based on each of the terms mentioned. “Spinal manipulation” is a term used frequently by health care providers but not the general public, so for the purpose of this study, the more inclusive terms “chiropractic” and “chiropractor” were also searched to access more general Twitter discourse. Important to note is that searching for tweets with the terms “chiropractic” and “chiropractor” also captured these terms prefixed with a hashtag (#), a key component of Twitter communication [32,47,55]. Data collection of each tweet included: username, twitter handle, tweet, number of retweets, number of likes, time and date, and city location (if the Twitter user had included geo-tags). The data collection resulted in the following number of tweets (including retweets): Corpus 1, “chiropractic,” 18,354 tweets; Corpus 2, “chiropractor,” 12,918 tweets; and Corpus 3, “spinal manipulation,” 67 tweets.

An initial exploratory analysis of approximately 600 tweets in each of Corpora 1 and 2, as well as the entire Corpus 3 was conducted in order to identify general themes in the discourse [56]. Next, spam was deleted from Corpora 1 and 2 by highlighting very active users (more than 15 tweets in the month) and removing accounts deemed to be bots, which are automated (robotic) accounts programmed to perform simple, repetitive tasks on social media. On Twitter, a bot might tweet to provide links to a wide range of promotional material, follow accounts that other accounts follow, retweet others’ tweets based on key words, or tweet nonsensical phrases with embedded key words. Bots typically have no bios, tweet extensively and periodically (eg, every hour), and are seldom followed by human users. In addition, because there were a large number of bots spreading promotional spam in the corpus, all usernames with the words “job” or “deal” were removed on the grounds of being promotional spam bots. Although spam does provide data worthy of analysis, real and active users with a large body of “followers” are much more influential [57]. Following the removal of most discernible spam and bots, the final number of tweets in each corpus is as followed: Corpus 1, “chiropractic,” 11,446 tweets; Corpus 2, “chiropractor,” 9182 tweets; and Corpus 3, “spinal manipulation,” 67 tweets.

Using a confidence level of 95% and a CI of 3.9, it was determined that a sample of 600 tweets, sequentially organized by date were to be analyzed in Corpora 1 and 2 (n=1200). All tweets were analyzed in Corpus 3 (n=67). Tweets were deemed skeptical or critical if they raised any doubts of efficacy, highlighted potential health risks, mentioned excessive pain, labeled the treatment with negative, derogatory terms, linked SMT to criminal activity or questionable health practices, or asked questions concerning efficacy that were, at times, followed by links. After all skeptical or critical tweets were identified, each tweet was analyzed with greater scrutiny (opening links, viewing emojies and pictures, and assessing the context of the dialogue) and was identified as explicitly containing skeptical or critical views of SMT. Because textual analysis is often understood to be a subjective process, 50% of the total tweets in the sample (n=600) were tested for inter-coder reliability using Cohen kappa, resulting in κ=.95. This Kappa score indicates almost perfect inter-rater agreement according to Landis and Koch’s benchmark standards [58].

To shed light on how the tweets were disseminated, the number of mentions and hashtags in each critical or skeptical tweet were counted. A mention is a Twitter tool whereby a tweet contains the “@” sign, followed by a username of another Twitter account. This user who is mentioned is notified of appearing in another person’s tweet. Using mentions often creates dialogue between twitter users. A hashtag, “#,” followed by a key word or phrase is a way to create an information category in which relevant information can be appended. Hashtags can be searched on Twitter to view all tweets constituting a particular category.

Next, the presence of skeptical or critical tweets were assessed by assembling lists of the top 10 retweets and liked tweets in each corpus. Assessing retweets and likes highlights how much attention particular tweets have been given and illustrates which information is most widely disseminated using Twitter tools. In addition, in order to explore how controversial applications of SMT are represented, all tweets containing “ADHD,” “immune system,” and “blood pressure” (all applications with evidence to support the use of SMT) were highlighted and examined. The objective was to determine if there were tweets critical of claims suggesting chiropractic or SMT can benefit ailments pertaining to these health issues. Finally, all tweets with the key words: “vertebral artery dissection” or “stroke,” were identified and analyzed, as these terms, highlighted in the relevant literature on risk, are indicative of potential risks associated with SMT [13,21,22].

## Results

**Figure 1:**
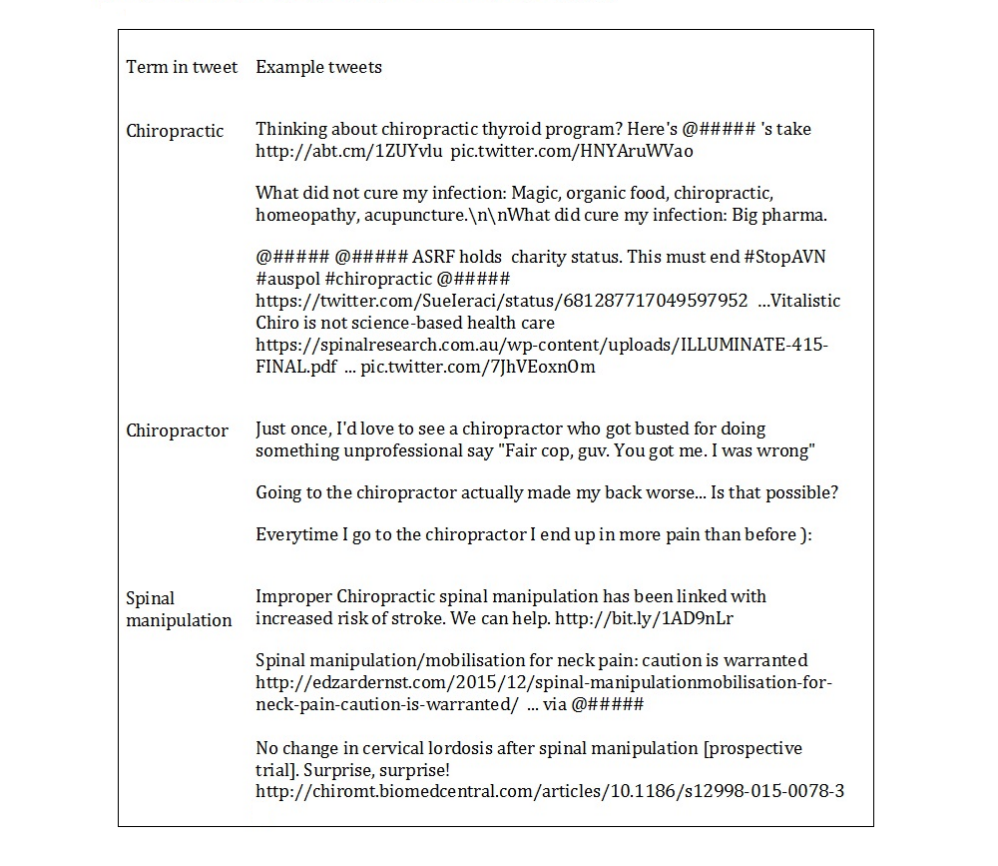
Examples of skeptical or critical tweets with usernames replaced by @#####.

Of all tweets analyzed in Corpora 1 and 2 (n=1200), a total of 77 tweets (6.42%), 95% CI (2.52%-10.32%) contained skeptical or critical sentiment. Following in-depth analysis, 25 of the 77 tweets contained explicitly skeptical or critical content, representing 2.08% of the more general Twitter discourse, 95% CI (0%-5.98%). In Corpus 3: “spinal manipulation” (n=67), 25 tweets, 37% of the corpus, contained skeptical or critical sentiments. Following in-depth analysis, 9 of the 25 tweets contained explicitly skeptical or critical content, representing 13% of the Corpus. Of the 34 total skeptical or critical tweets, a total of 7 contained mentions (21%) and 5 contained hashtags (15%). For examples of skeptical or critical tweets, refer to Figure 1. To view all skeptical or critical tweets, refer to Multimedia Appendix 1.

**Figure 2:**
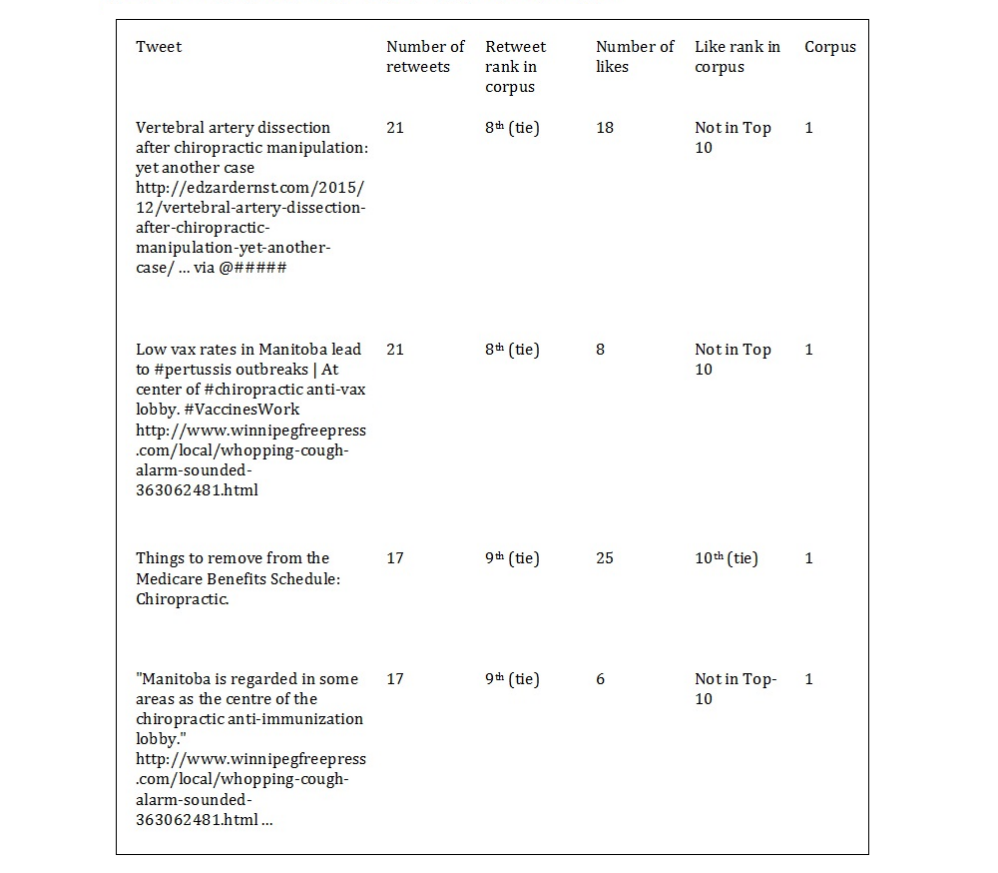
Skeptical or critical tweets ranking in the top 10 for retweets and likes.

Regarding benefits for specific health issues, a total of 88 tweets of 20,695 mentioned the terms “ADHD,” “immune system,” or “blood pressure.” Of those 88 tweets, 4 (5%) were identified as skeptical or critical (Table 1). These 4 tweets can be found in the Multimedia Appendix 1. In terms of highlighting specific risks associated with SMT, of 20,695 tweets, 30 (0.14%) mentioned “stroke” or “vertebral artery dissection.” Of these 30 tweets, 22 (73%) contained explicitly skeptical or critical content (Table 2). Of these 22 tweets, all unique skeptical or critical tweets can be found in the Multimedia Appendix 1. Finally, regarding tweet impact in their respective Corpus, 4 skeptical or critical tweets ranked in the top 10 for retweets, whereas 1 skeptical or critical tweet ranked in the top 10 for likes (Figure 2). All these tweets were found in Corpus 1.

**Table 1:** Tweets and skeptical or critical tweets for health issues.

Health issue term	Tweets containing term	Skeptical or critical tweets containing term
Attention deficit hyperactivity disorder	18	3
Immune system	44	1
Blood pressure	26	0
Total	88	4

**Table 2:** Tweets and skeptical or critical tweets highlighting risks of spinal manipulation therapy.

Term associated with risk	Total tweets containing term	Skeptical or critical tweet containing term
Stroke	10	4
Vertebral artery dissection	20	18
Total	30	22

## Discussion

### Principal Findings

This analysis of social media finds that the efficacy of chiropractic and SMT is rarely doubted or questioned on Twitter. In addition, the potential risks are rarely mentioned or debated. The manner in which efficacy and risks are tweeted across the 3 corpora, based on the different search terms, however, reveals some insights regarding how contentious or contrary information is, or can be, disseminated on the social media platform.

“Spinal manipulation” is a more specialized term and as a result seems likely to be associated with more technical twitter discussions. As evident in Multimedia Appendix 1, of the 9 skeptical or critical tweets using “spinal manipulation” include a link to academic studies or research. Tweeters using this term, therefore, seem more aware of the debates surrounding the efficacy and potential risks of SMT.

Although both terms “chiropractor” and “chiropractic” yielded very few skeptical or critical tweets, some of the skeptical or critical tweets using “chiropractic” had been liked and retweeted significantly. This suggests that some skeptical or critical perspectives have an impact on the tweeting public despite the fact that their voices are marginal in number. A question arises, however, as to whether this information is simply being liked and retweeted by like-minded individuals inside of a social network bubble or if this information is reaching new audiences [48]. Even the discussions around highly controversial uses of SMT—as highlighted by our analysis of the Tweets associated with asthma, ADHD, and the immune system—did not generate significant critical attention.

Studies have shown that group polarization is prevalent in Twitter conversations involving politics and contentious issues [44,45,59], thereby limiting information dissemination among those with opposing views. It is yet to be explored, however, as to whether something similar occurs in health-related discourse on Twitter. If it is the case that the increasingly personalized algorithms structuring the Internet expose individuals more often to information that reinforces one’s view and less often to novel information [48], it’s possible that heuristics like the confirmation bias are being magnified [51-53]. The degree to which information is shared among dissimilar individuals on social media regarding less politicized topics, such as health, still requires further research [59]. Regardless, on Twitter, studies have shown that hashtags and mentions can prove to be useful tools for disseminating information more widely and for engaging more diverse audiences [45,47]. In our study, of the 34 tweets explicitly expressing skepticism or doubt in the sampled tweets, only 7 included mentions and 5 included hashtags. This arguably demonstrates a narrow scope of information dissemination. In short, those in the health community wishing to make their critiques of chiropractic and SMT better known to a broader public might find using mentions and hashtags beneficial to their cause—especially because of the impact that Twitter can have on the formation of views is well-documented [35,36].

### Limitations

This study has several limitations worth noting. Given the nature of Twitter discussions and the somewhat limited access provided by Twitter’s API, it can be challenging to capture a comprehensive collection of tweets on any topic. In addition, other potential terms such as “chiro” and “spinal adjustment” are present on Twitter, which may produce datasets with somewhat different results. Finally, although December 2015 was chosen at random, there is nothing to suggest that other time frames would be significantly similar or different. Despite these limitations, this study highlights the degree to which discussions of risk and critical views on efficacy are almost completely absent from Twitter.

### Conclusion

In total, Twitter representations of SMT and chiropractic are overwhelmingly not skeptical or critical. The ongoing debates regarding efficacy and risk in the academic literature and the popular press [60-62] do not have a strong presence on Twitter. This study provides insight into how Twitter users discuss SMT and chiropractic and suggests that, in the aggregate, the information on this social media platform is far from balanced or informed. Although voices do exist which raise concerns of SMT efficacy and highlight potential risks associated with the practice, their presence is marginal in overall Twitter discourse.

## Appendix

Multimedia Appendix 1 All skeptical and critical tweets.

## Abbreviations

ADHD: attention deficit hyperactivity disorder
API: application programming interface
SMT: spinal manipulation therapy
