# Supplementary material for: Chiropractic and Spinal Manipulation Therapy on Twitter: Case Study Examining the Presence of Critiques and Debates
Source: JMIR Public Health Surveill. 2016 Sep 16;2(2):e153. doi: 10.2196/publichealth.5739 (PMC5045521; doi:10.2196/publichealth.5739)
Supplement: Multimedia Appendix 1 [file publichealth_v2i2e153_app1.pdf]

Appendix: Skeptical/critical tweets

All user names in mentions have been replaced by @##### to protect users' privacy

Table 1: All 7 tweets with explicit skeptical/critical content using the term 'chiropractic'

A question from our inbox:\n\n"Did anyone find that chiropractic care during their pregnancy was integral to the... http://fb.me/2jjnWls1d
Chiropractic and Acupuncture? Learn why medical doctors are afraid of these therapies. http://ln.is/annalaurabrown.com/Y9yPQ …\nacupuncture/
Thinking about chiropractic thyroid program? Here's @##### 's take http://abt.cm/1ZUYvlu pic.twitter.com/HNYAruWVao
A chiropractic adjustment that went horribly wrong. #ExplainAFilmPlotBadly pic.twitter.com/hXYxKbtIyY
What did not cure my infection: Magic, organic food, chiropractic, homeopathy, acupuncture.\n\nWhat did cure my infection: Big pharma.
.@#####@##### ASRF holds charity status. This must end #StopAVN #auspol #chiropractic @#####https://twitter.com/SueIeraci/status/681287717049597952 …Vitalistic Chiro is not science-based health care https://spinalresearch.com.au/wp-content/uploads/ILLUMINATE-415-FINAL.pdf … pic.twitter.com/7JhVEoxnOm
@##### @##### @##### This #chiropractic org holds charity status, based on magic #auspol #health https://twitter.com/SueIeraci/status/681286733313343488 …Rational Chiros - is it time for a split with the vitalists? https://spinalresearch.com.au/wp-content/uploads/ILLUMINATE-415-FINAL.pdf … pic.twitter.com/pQ82wnnLwN

Table 2: All 18 tweets with explicit skeptical/critical content using the term 'chiropractor'

Just once, I'd love to see a chiropractor who got busted for doing something unprofessional say "Fair cop, guv. You got me. I was wrong"
In what world does medical attention for a doctor's diagnosis mean needing to go to the chiropractor because he's kind of like a doctor. No
Getting x-rayed at the chiropractor in a little bit and they're probably gonna make me pay before they tell me what's wrong
Going to the chiropractor actually made my back worse... Is that possible?
Gotta love when you see the chiropractor and a week later your back hurts again
That recognition of a vertebral artery dissection by chiropractor was published as a case report speaks volumes. https://twitter.com/smperle/status/674921971537301504 …Chiropractic Response to a

Spontaneous Vertebral Artery Dissection http://www.journalchiromed.com/article/S1556-3707(15)00090-5/abstract?elsca1=etoc&elsca2=email&elsca3=1556-3707_201509_14_3_&elsca4=Physical%20Medicine%20and%20Rehabilitation …
Realised I can no longer move my neck vertebrae but chiropractor visits are slowly causing every muscle in my body to die
Chiropractor is doing his absolute best to help but I guess some things can't be fixed this way
Everytime I go to the chiropractor I end up in more pain than before):
I kinda feel like the chiropractor raped my spine. #spinalrape
When you can't parse evidence: GREAT WILL YOU DEBATE MY DAD WHOS A CHIROPRACTOR--that's related to science, right? https://twitter.com/lalaruefrench75/status/677671735144128516 …@##### Perfect! Then I'd like to offer you an invitation to publicly debate an MD on vaccine safety @##### @##### @#####
I really need to go to a chiropractor but I refuse to have to rely on going.. Cause once you start you cannot stop
went to the chiropractor this morning and now I'm laying in bed and my back is killing me
so i went to the chiropractor for my back but he cracked my toes wtf
my cousin is a chiropractor and he just gave me an adjustment and I swear my left arm is numb
when the chiropractor makes your back worse, like you had one job man
If you want to be rich be a chiropractor they are the biggest thief's in the world
@##### That sucks : /\n\nBut don't see a chiropractor because those are dangerous quacks.

Table 3: All 9 tweets with explicit skeptical/critical content using the term ‘spinal manipulation’

Lordosis does not change with spinal manipulation http://chiromt.biomedcentral.com/articles/10.1186/s12998-015-0078-3
Improper Chiropractic spinal manipulation has been linked with increased risk of stroke. We can help. http://bit.ly/1AD9nLr
Does cervical lordosis change after spinal manipulation for non-specific neck pain? A prospective cohort study http://chiromt.biomedcentral.com/articles/10.1186/s12998-015-0078-3
No change in cervical lordosis after spinal manipulation [prospective trial]. Surprise, surprise! http://chiromt.biomedcentral.com/articles/10.1186/s12998-015-0078-3
New paper on the mechanism of spinal manipulation. Read about it here!\n http://ow.ly/Vzq4S
#Chiropractic or #Osteopathic Spinal manipulation/mobilisation for neck pain: caution is warranted http://edzardernst.com/2015/12/spinal-manipulationmobilisation-for-neck-pain-caution-is-warranted/ … via @#####

Does cervical lordosis change after spinal manipulation for non-specific neck pain? A… https://chiroresearcher.wordpress.com/2015/12/07/does-cervical-lordosis-change-after-spinal manipulation-for-non-specific-neck-pain-a-prospective-cohort-study … pic.twitter.com/BU0MRvapdV
Spinal manipulation/mobilisation for neck pain: caution is warranted http://edzardernst.com/2015/12/spinal-manipulationmobilisation-for-neck-pain-caution-is-warranted/ … via @#####
YAY! Mainly focuses on the nervous system as spinal manipulation directly affects it, but all systems are evaluated. https://twitter.com/ashynoodles/status/67212090459645952 …What systems of the body do they target most ? https://twitter.com/mrgrigsby_/status/672119480345079810

Table 4: 4 skeptical/critical tweets with health terms 'ADHD', 'immune system', 'blood pressure'

.@##### I am not a fan. There is no evidence that chiropractic care is effective for #ADHD.
I'd also have been a very, very successful Amway distributor or "chiropractic neurologist" curing ADHD & autism https://twitter.com/independent/status/675405974501363712 …11 alternative careers Donald Trump would have been better at http://i100.io/gqjnIge pic.twitter.com/RovZbAwV1j
@##### This is why I once almost had to fight a chiropractor. That and he said he could cure my ADHD.
Okay, you've got to a chiropractor who told her that her not being ill for two years was proof her immune system wasn't working.

Table 5: All unique tweets highlighting risks of SMT with terms 'stroke' or 'vertebral artery dissection' (excluding replicated retweets)

Chiropractic neck manipulation can cause stroke http://edzardernst.com/2015/01/chiropractic-neck-manipulation-can-cause-stroke/ … via @#####
Improper Chiropractic spinal manipulation has been linked with increased risk of stroke. We can help. http://bit.ly/1AD9nLr
1 year ago today I was nearly killed after getting my neck adjusted at a chiropractor. A stroke. 3 days ICU and 7 days in a rehab hospital.
Do *NOT* let anyone (chiropractor, massage therapist, etc…) perform cervical (neck) manipulations on you. Dangerous. https://twitter.com/EdzardErnst/status/677182079327715328 …Vertebral artery dissection after chiropractic manipulation: \nyet another case \n\nhttp://edzardernst.com/2015/12/vertebral-artery-dissection-after-chiropractic-manipulation-yet-another-case/ … via @#####
Vertebral artery dissection after chiropractic manipulation: yet another case #chirokills #precautionaryprinciple http://ow.ly/WucHo
Vertebral artery dissection after chiropractic manipulation: yet another case http://edzardernst.com/2015/12/vertebral-artery-dissection-after-chiropractic-manipulation-yet-another-case/ … via @#####
Not everone will dodge those vertebral artery dissection bullets

https://www.facebook.com/1375720392732602/photos/a.1375737219397586.1073741827.1375720392732602/1504839606487346/?type=3&theater … #SinghBCA #chiropractic
Scepticism! Vertebral artery dissection after chiropractic manipulation: yet another case | Edzard Ernst: Whil... http://bit.ly/1QoXcc5
Vertebral artery dissection after #chiropractic manipulation: yet another case http://edzardernst.com/2015/12/vertebral-artery-dissection-after-chiropractic-manipulation-yet-another-case/ … via @#####
